# Development of electron ptychography from algorithms, detectors to its applications

**DOI:** 10.1186/s42649-025-00119-1

**Published:** 2025-12-03

**Authors:** Wonwoo Suh, Jeewon B. Choi, Keun-Yeol Park, Celesta S. Chang

**Affiliations:** 1 Department of Physics and Astronomy, Seoul National University, Seoul, 08826 Republic of Korea; 2 Institute of Applied Physics, Seoul National University, Seoul, 08826 Republic of Korea

**Keywords:** Four-dimensional scanning transmission electron microscopy (4D-STEM), Electron ptychography, Multislice electron ptychography (MEP), Phase retrieval algorithm, Direct detector

## Abstract

Electron ptychography has emerged as a powerful computational imaging technique using four-dimensional scanning transmission electron microscopy, greatly exceeding the resolution limits of conventional electron microscopes by quantitative phase retrieval. This paper presents recent algorithmic developments and technological requirements for detectors used in electron ptychography, as well as applications in different fields of nanoscience. The application range covers high-resolution imaging of beam-sensitive specimens, light element detection, and three-dimensional reconstruction, making electron ptychography a versatile technique for materials characterization.

## Introduction

Direct imaging of individual atoms has long been a fundamental goal to aid the understanding of various scientific phenomena. Among the available experimental tools, transmission electron microscopy (TEM) is a uniquely powerful technique, now capable of resolving atomic structures at or below sub-angstrom precision. Historically, the efforts to achieve higher resolution were primarily focused on reducing the electron beam wavelength by using higher accelerating voltages in the first five decades. High-voltage electron microscopes (HVEM) operating at 1–3 MeV appeared in the mid-1960s boasting atomic resolution (~ 0.1 nm) (Egerton 2014; Takaoka et al. 2000), however such high voltages posed significant limitations, most importantly in the form of increased beam damage to specimens. As an alternative, intermediate voltage electron microscopes (IVEM) with accelerating voltages of 200–400 keV offered a compromise between resolution and sample preservation (Zhou and Chiu 1993). To compensate for poor resolution compared to HVEMs, short focal length objective lenses were designed and the electron optical stability was improved, reducing the effect of chromatic and spherical aberrations (Egerton 2014). Nonetheless, the intrinsic lens aberrations of electromagnetic systems remained a fundamental limiting factor, until the advent of spherical aberration (Cs) correctors which marked a pivotal advance in the evolution of atomic-resolution TEM (Haider et al. 1998). These developments not only enhanced the capabilities of conventional TEM, but—when later adapted to scanning transmission electron microscopy (STEM), originally introduced in the 1960 s (Crewe et al. 1969)—enabled STEM to achieve sub-angstrom resolution and analytical performance previously unattainable in earlier configurations (Batson et al. 2002).

While both high-resolution TEM (HRTEM) and STEM can achieve atomic resolution, they use fundamentally different imaging modes. HRTEM relies on phase contrast formed by a wide, parallel beam making it highly sensitive to imaging conditions such as defocus, lens aberrations and sample thickness. In contrast, STEM image is formed by scanning a focused probe across the sample and integrating scattered electrons at each probe position typically using annular dark-field (ADF) detectors. This provides higher resolution and robust Z-contrast with minimal effects from defocus and sample thickness variations (Pennycook and Jesson 1991). Although STEM is often preferred for its relatively straightforward analysis compared to TEM, it no longer retains phase information due to incoherent detection, imposing a fundamental limit on attainable resolution (Rodenburg 1989). In contrast, four-dimensional STEM (4D-STEM) records a full two-dimensional (2D) diffraction pattern produced at each probe position in a 2D scan in real space which presents full complex scattering information of the sample (Ophus 2019). Also, the full diffraction dataset allows for virtual diffraction imaging, given the freedom to select the integration angles beyond conventionally used detectors with fixed geometries. Most notably, the overlapping diffraction patterns in a 4D dataset could be used for ptychographic phase reconstruction, further extending the resolution beyond conventional STEM limits.

**Fig. 1:**
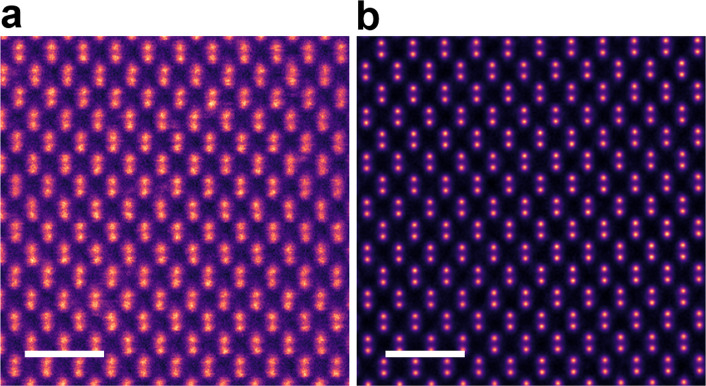
Comparison between conventional STEM imaging and ptychographic reconstruction of silicon viewed in the (110) direction. **a** High-angle annular dark-field (HAADF) image showing dumbbell-like atomic columns in silicon, but with limited resolution. **b** Ptychographic phase reconstruction from the same region, revealing enhanced spatial resolution and improved contrast of atomic columns. Scale bars are 1 nm

First introduced in the context of optical and X-ray imaging (Thibault et al. 2008), ptychography is a phase retrieval technique that has recently been adapted to electron microscopy. Rather than integrating a fraction of the scattered signal to form an image as in STEM imaging, it works by computationally reconstructing the specimen’s complex transmission function – a quantity that encodes both phase and amplitude information – using a set of diffraction patterns. During a raster scan, a convergent electron beam illuminates partially overlapping regions of the specimen, producing diffraction patterns that contain information from shared areas, thereby creating an interdependent 4D dataset that must be jointly analyzed. Solving for a single, self-consistent transmission function hence requires a dedicated, robust computational algorithm. Once reconstructed, it provides unprecedented spatial resolution as in Fig. 1, showing stark contrast in resolution compared to conventional STEM imaging. Furthermore, the methodology using 4D-STEM for electron ptychography offers its extension to applications such as low-dose imaging and robust visualization of weakly scattering structures such as light elements, strain fields, and interfaces (Jiang et al. 2018), which will be discussed later in this paper.

This review provides a concise overview of the principles, capabilities, and recent advances in electron ptychography. The first section introduces the theoretical foundations of ptychographic phase reconstruction, including key algorithmic approaches, and extensions such as the multislice formalism for handling thicker specimens. The second section addresses practical considerations in implementation, with the focus on the development of high-speed, high-dynamic-range detectors designed to meet the specific demands of high-resolution electron ptychographic acquisition. Lastly, we highlight emerging applications in various fields where electron ptychography offers distinct advantages over conventional techniques. Altogether, this review aims to provide a clear overview with a current perspective on how electron ptychography is evolving into a versatile and quantitative tool for high-resolution electron microscopy.

## Advances in electron ptychography algorithms

**Fig. 2:**
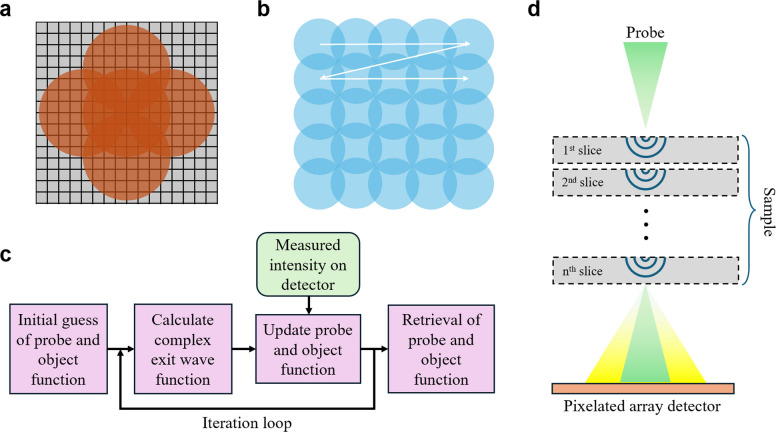
Schematic overview of the scanning geometry, phase retrieval concept, and algorithmic framework in ptychography. **a** Phase information of the exit wave can be retrieved from the overlapping regions of the recorded diffraction patterns in the detector plane. **b** The image shows probe positions in a typical raster scan in real space, where iterative ptychography uses two or more overlapping scanning regions. **c** Basic flowchart of an iterative ptychography algorithm. **d** Multislice electron ptychography divides the sample into arbitrary slices and calculates wave propagation through each slice

The conceptual foundation for ptychographic reconstruction originated from coherent diffractive imaging (CDI), in which phases can be retrieved from a continuous diffraction pattern oversampled beyond the Nyquist interval (Miao 2025). In 1969, Hoppe and Hegerl (Hegerl and Hoppe 1970) extended the concept of CDI by proposing the idea that complex wave information can be retrieved from the interference in the overlapping regions of diffraction spots (Fig. 2a). Later, the Gerchberg and Saxton (Gerchberg and Saxton 1971, 1972) formalized an iterative phase retrieval algorithm combining the interference patterns with a priori known constraints in real space. This was refined once more by Fienup (Fienup 1978, 1982) through incorporating a loosened real-space constraint, improving stability for practical experimental conditions. Building upon the foundations of CDI, ptychography emerged as a more advanced phase retrieval technique that utilizes multiple diffraction patterns while rastering the probe across the specimen. A variety of ptychography algorithms have been proposed over the years, which can be classified differently. While some classifications consider whether the algorithms are designed to address experimental uncertainties such as partial coherence, noise, and position errors (Rodenburg and Maiden 2019; Clark et al. 2503), it is often classified into non-iterative and iterative approaches based on their reconstruction methods.

Representative algorithms for non-iterative approaches, also called direct methods, include single sideband (SSB) ptychography (Rodenburg et al. 1993) and Wigner distribution deconvolution (WDD) (Rodenburg and Bates 1992). SSB ptychography exploits the double-overlap regions in reciprocal space to directly reconstruct phase information. It offers fast and intuitive reconstruction with good noise tolerance. However, it is mainly suitable for thin, weak phase specimens such as graphene, and it is hard to apply for thicker materials where the weak phase approximation no longer holds. WDD applies Wiener filtering in the Wigner distribution domain for deconvolution-based recovery enabling high resolution reconstruction. However, it is computationally intensive and sensitive to noise compared to SSB ptychography. In addition, it requires diffraction patterns that are oversampled in reciprocal space, at or beyond the Nyquist condition of the full-field of view.

In contrast, iterative approaches include Ptychographic Iterative Engine (PIE), extended PIE (ePIE), Difference Map (DM), Relaxed Averaged Alternating Reflections (RAAR), and Maximum Likelihood (ML) methods, etc. One of the key features of iterative ptychography algorithms is that the scanning probe positions are intentionally overlapped in real space, and this overlap imposes additional contraints across the dataset without requiring explicit overlap conditions in reciprocal space (Fig. 2b). By exploiting these real space overlaps, most iterative algorithms update the probe and object functions by utilizing the measured intensity (Fig. 2c). PIE (Rodenburg and Faulkner 2004) reconstructs the object function iteratively by using a series of diffraction patterns acquired at different probe positions in real space. The ePIE (Maiden and Rodenburg 2009) updates both the probe and object functions during the iteration process, which has been successfully applied in practical electron ptychography (Jiang et al. 2018). Subsequent developments such as DM (Gravel and Elser 2008) and RAAR (Luke 2004) aim to improve convergence speed and robustness by formulating the problem in terms of projection operators or reflection maps. The ML (Thibault and Guizar-Sicairos 2012) method reformulates ptychographic reconstruction as an optimization problem in probabilistic terms, providing a powerful way to incorporate experimental uncertainties like statistical noise. Later, a mixed-state approach was integrated into the ptychographic algorithm to model partial coherence and other decoherence effects as well (Thibault and Menzel 2013).

Earlier algorithms relied mainly on weak-phase approximation, which assumes minimal phase distortion of the electron wave as it passes through the sample. This made atomically thin two-dimensional (2D) materials an ideal subject for electron ptychography. In 2018, Jiang et al. successfully applied electron ptychography to a twisted bilayer MoS_2_ with a thickness of 9.8 Å, demonstrating practical feasibility of the algorithm on 2D materials (Jiang et al. 2018). On the other hand, performing electron ptychography on thicker samples was confronted with challenges due to multiple scattering and associated dynamical effects. Before ptychography, multislice method was first introduced in 1957 to fully understand electron wave propagation inside a material while accounting for multiple scattering in thick samples (Cowley and Moodie 1957). It works by dividing the sample into multiple slices and propagating the wavefront sequentially through each slice (Fig. 2d), which has been widely used until now to simulate HRTEM and STEM images of thick samples (Maiden et al. 2012). Such approach was later incorporated into ptychographic algorithms, leading to the development of multislice electron ptychography (MEP) enabling successful reconstruction of samples with greater thickness and structural complexity (Chen et al. 2021; Dong et al. 2024).

The development of various ptychographic algorithms, together with pixelated direct electron detectors capable of recording full diffraction patterns at each probe position, brought significant enhancement of resolution, far exceeding the so-called “sub-angstrom” resolution achievable with traditional hardware-based aberration corrected microscopes. Jiang et al. applied ePIE algorithm on MoS₂ and achieved a record-breaking spatial resolution of 0.39 Å (Jiang et al. 2018; Rodenburg 2018). Later, Chen et al. utilized the ML algorithm to further push the spatial resolution to 0.23 Å (Chen et al. 2021). In addition to improvements in lateral resolution, depth-resolved imaging became possible through MEP reconstruction. Unlike conventional STEM which averages signals from several unit-cells to yield a 2D projected image, we now have access to see each unit-cell slice by slice, effectively enabling three-dimensional imaging of the specimen. Using this approach, Chen et al. achieved a depth resolution of 2.7 nm, illustrating its capability to detect interstitial atoms in garnet oxides and highlighting the technique’s ability to capture subtle features in complex materials (Chen et al. 2407).

## Direct detectors for electron ptychography

A key technological breakthrough that transformed electron ptychography from a conceptual approach into a practical STEM imaging technique was the advent of direct electron detectors as shown in.

**Fig. 3:**
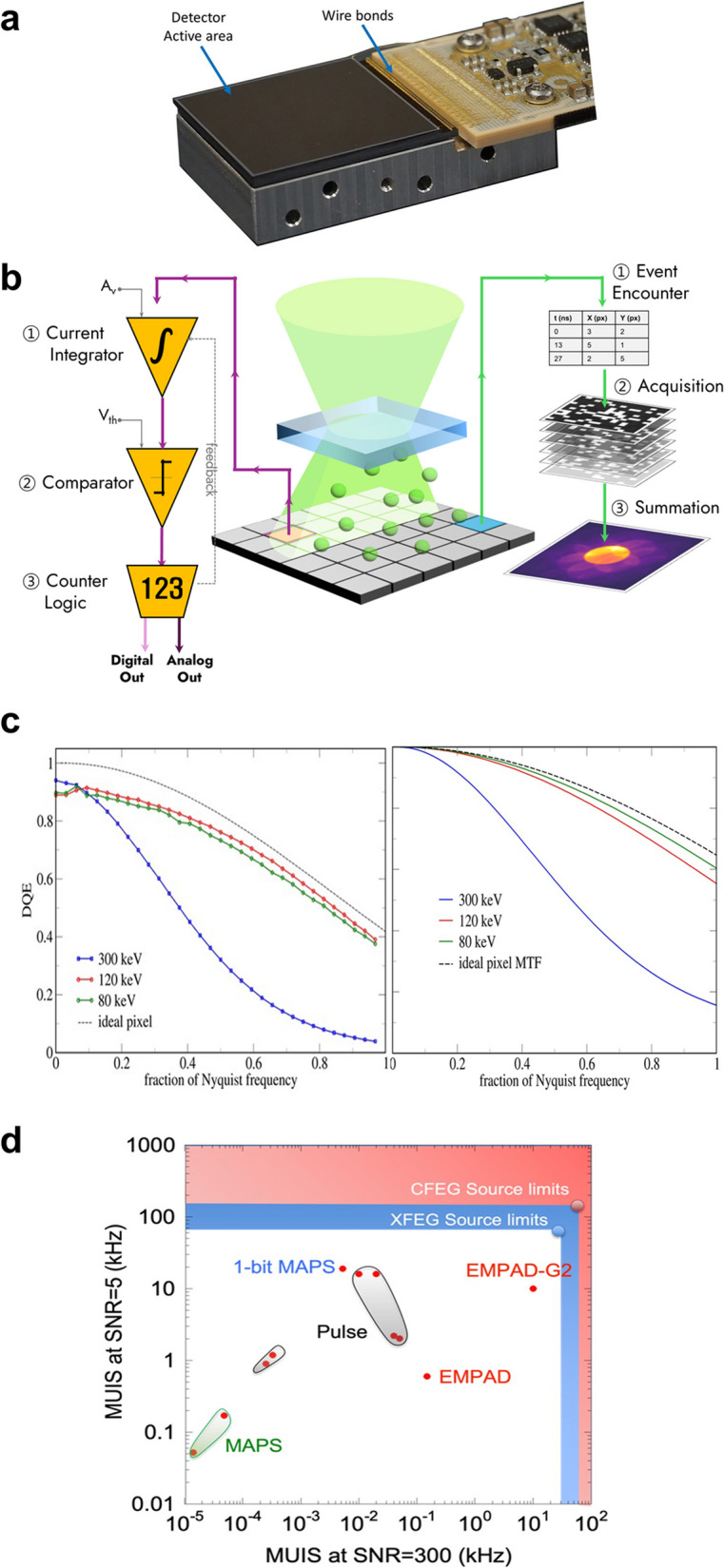
Factors to consider in detectors for electron ptychography. **a** Photograph of an EMPAD-G2 pixelated detector, showing the active sensor area and the integrated circuit wire-bonded to the sensor. **b** Schematic comparison between current-based and event-based detection architectures. The current-based approach integrates charge over time, while the event-based detector counts individual electron events, highlighting the fundamental differences in signal processing mechanisms. **c** Typical detector performance plots for (left) detector quantum efficiency (DQE) and (right) modulation transfer function (MTF), plotted over different acceleration voltages and spatial frequency. **d** Overview of various detector architectures in terms of their maximum usable imaging speed (MUIS) at different signal-to-noise ratio (SNR) levels. Panels **a**, **c**, and **d** are adapted from Ref. (Philipp et al. 2022)

Figure 3a, which is mounted below the sample and enables the recording of full diffraction patterns at each probe position (Ophus 2019). In particular, advances in monolithic active pixel sensors (MAPS) and hybrid pixel array detectors (PADs) have provided a compelling alternative to traditional charge-coupled device (CCD) cameras, offering improvements in speed, sensitivity, and dynamic range (Levin 2021). As ptychographic reconstruction algorithms began to exploit oversampled diffraction data to achieve unprecedented resolution, the demands placed on detector performance also increased, requiring faster acquisition, finer pixel sampling, and an expanded dynamic range to capture both the intense central beam and weak high-angle scattering simultaneously. In this section, we outline such essential detector characteristics required for high-performance 4D-STEM electron ptychography and highlight recent state-of-the-art pixel detectors that have been successfully applied in cutting-edge experiments.

A primary requirement for detectors used in 4D-STEM electron ptychography is the wide dynamic range. Ptychographic reconstruction depends on both the intense central beam with currents of tens to hundreds of pico-amperes per pixel, and weak high-angle scattered signals including single-electron events at the periphery of the diffraction pattern. Therefore, detectors must record variations in electron intensity spanning several orders of magnitude within a single frame without suffering from saturation or underflow artifacts (Li et al. 2022). To accommodate such a broad range of signals, most direct detectors adopt one of two strategies: event-based counting or current-based integration (Fig. 3b). Event-based detectors register and digitally count each electron or X-ray. This approach works well for low-dose imaging, where events are temporally sparse, but it can lose accuracy under high-dose conditions due to signal pile-up. By contrast, current-based detectors integrate the total charge over a set time interval, making them more suitable for high-dose experiments with stronger beams. However, their performance depends on collecting the entire deposited energy within the detector pixel. Particularly in thin sensors, significant energy straggling leads to a Landau distribution of deposited energy introducing noise that is difficult to average out, thereby necessitating careful optimization of sensor thickness especially at high acceleration voltages (Philipp et al. 2022; Tate et al. 2016).

In addition to dynamic range, frame rate is another critical factor for effectively recording diffraction patterns for 4D-STEM electron ptychography. To make 4D-STEM acquisition practically comparable to conventional STEM imaging in terms of acquisition time, detectors must operate hundreds to thousands of frames per second at megapixel resolutions. For example, to complete a full 4D-STEM scan recording of 128 × 128 pixel diffraction patterns over a 1024 × 1024 pixel image area within 5 min, the detector must sustain a frame rate of approximately 3.5 kHz or higher. Recent advances in high-speed pixelated detectors now enable frame rates exceeding 1 kHz, allowing data collection at conventional STEM scanning speeds and facilitating phase and ADF imaging under identical optical conditions. Moreover, high frame rates also serve to suppress stage drift and sample vibrations during acquisition, while minimizing radiation damage due to shortened exposure times. However, increasing frame rate often comes at the cost of reduced signal-to-noise ratio (SNR) or increased readout noise, and detector design must strike a balance between speed, dynamic range, and noise performance. To better quantify this trade-off, recent studies have even introduced the concept of maximum usable imaging speed (MUIS), which defines the highest frame rate at which a target SNR can still be reliably achieved. (Philipp et al. 2022).

Furthermore, accurate detection of spatial frequencies near the detector’s resolution limit requires both high detection sensitivity and precise signal localization. The performance of a detector in registering incident electrons is commonly evaluated using parameters such as detective quantum efficiency (DQE) and modulation transfer function (MTF). DQE, defined as the square of the ratio between output and input SNRs, quantifies how efficiently a detector converts incident electrons into usable signals. MTF, on the other hand, describes the spatial frequency response of the detector. Ideally, a perfect detector should exhibit a DQE of unity across the entire spatial frequency range, and an MTF that remains flat up to the Nyquist limit. In practice, however, both DQE and MTF exhibit non-ideal characteristics that reflect inherent trade-offs in detector design. The MTF typically decreases with increasing spatial frequency due to signal spreading mechanisms, while the DQE is additionally suppressed by noise aliasing, particularly near the Nyquist frequency (Fig. 3c). More importantly, a high MTF at all spatial frequencies is not inherently desirable; under certain conditions, a modest reduction in high-frequency MTF—when caused by deterministic blur—can actually improve the DQE by suppressing aliased noise without disproportionately damping the high-frequency signal. This frequency-dependent behavior of DQE introduces a trade-off between spatial resolution and field of view. To access high-resolution information with a sufficient SNR especially at high beam energies such as 300 keV where delocalization becomes significant, one must employ higher magnification, which inevitably reduces the effective field of view. Consequently, optimizing detector performance for specific imaging conditions requires careful balancing of resolution, DQE, and dose efficiency, particularly in the context of dose-limited applications such as cryogenic or beam-sensitive materials. (McMullan et al. 2009; Chatterjee et al. 2021).

A variety of detectors have emerged to meet such demands of 4D-STEM electron ptychography, focusing on improvements in dynamic range, operating speed, and zero dead time performance. One such approach, a current-integrator-type pixel array architecture, has been implemented in the EMPAD-G2 (Electron Microscope Pixel Array Detector Generation 2), developed at Cornell. Based on the architecture of the first-generation EMPAD—now commercially available through Thermo Fisher—, the G2 advances key performance metrics with a mixed-mode design combining in-pixel current integration and digital overflow counting. (Philipp et al. 2022; Tate et al. 2016) A near-zero-dead-time readout scheme, realized through two parallel detector modules that alternate between signal acquisition and readout, allows continuous data capture even during frame transfer. This enables the detector to sustain frame rates up to 10 kHz with a near-unity duty cycle, a major improvement over the first generation which was limited to 1.1 kHz and suffered from sharp duty cycle degradation above that range. With its extended dynamic range (> 10⁶:1) and virtually uninterrupted acquisition, the G2 is well-suited for fast, high-fidelity imaging across samples with wide diffraction intensity variations.

A hybrid-pixel detector design that utilizes single-electron counting, offering a distinct approach from current integrator designs, is exemplified by the Arina system developed by Dectris. It provides high-speed readout up to 120,000 fps, with acquisition times on the order of a few microseconds per 192 × 192 diffraction pattern frames. The detector boasts a wide dynamic range, capable of accurately measuring from single electron events up to fluxes corresponding to approximately 10 pA of current. To maintain accurate electron counting at such high rates, the ARINA incorporates DECTRIS’s Instant Retrigger® technology. This method minimizes pulse pile-up by quickly re-evaluating signals within adjustable dead times after each detected event, preventing counting paralysis and improving count-rate correction. As a result, it delivers enhanced data quality even under intense electron flux. ARINA has shown promising SNR and angular fidelity (Stroppa et al. 2023), also enabling advanced acquisition modes such as synchronized acquisition with conventional ADF detectors enabled by its high frame rate (Seifer and Elbaum 2023).

Another event-driven detector architecture is implemented in Timepix3, which builds upon the Medipix3 photon-counting pixel technology. Each pixel records the arrival time and energy of individual electrons with nanosecond resolution (Poikela et al. 2014). This enables continuous, shutterless acquisition and enables dynamic sparse-beam electron ptychography. By taking advantage of event sparsity especially prominent at low-dose microscopy, Timepix3 reduces data readout bottlenecks and allows rapid acquisition without the dead time generally associated with current-based detectors (Jannis et al. 2022). As such, it excels in ultralow-dose imaging and is particularly suited for beam-sensitive materials, where both low electron counts and high temporal resolution are critical (Motúz et al. 2024; Bromberger et al. 2024).

State-of-the-art detectors for 4D-STEM electron ptychography have made significant progress in achieving wider dynamic range, faster imaging speeds, and efficient signal acquisition at higher spatial frequencies. Nevertheless, as pointed out by Phillip et al., the primary limiting factor for achieving high SNR at high imaging speeds remains the detector, rather than the electron source. As illustrated in.

Figure 3d, the maximum usable imaging speed (MUIS) at a given SNR for modern detectors still falls short of the limits imposed by thermal and cold field emission guns (FEGs), suggesting that further advancements in detector technology are both necessary and possible.

## Extension of ptychography to application fields

**Fig. 4:**
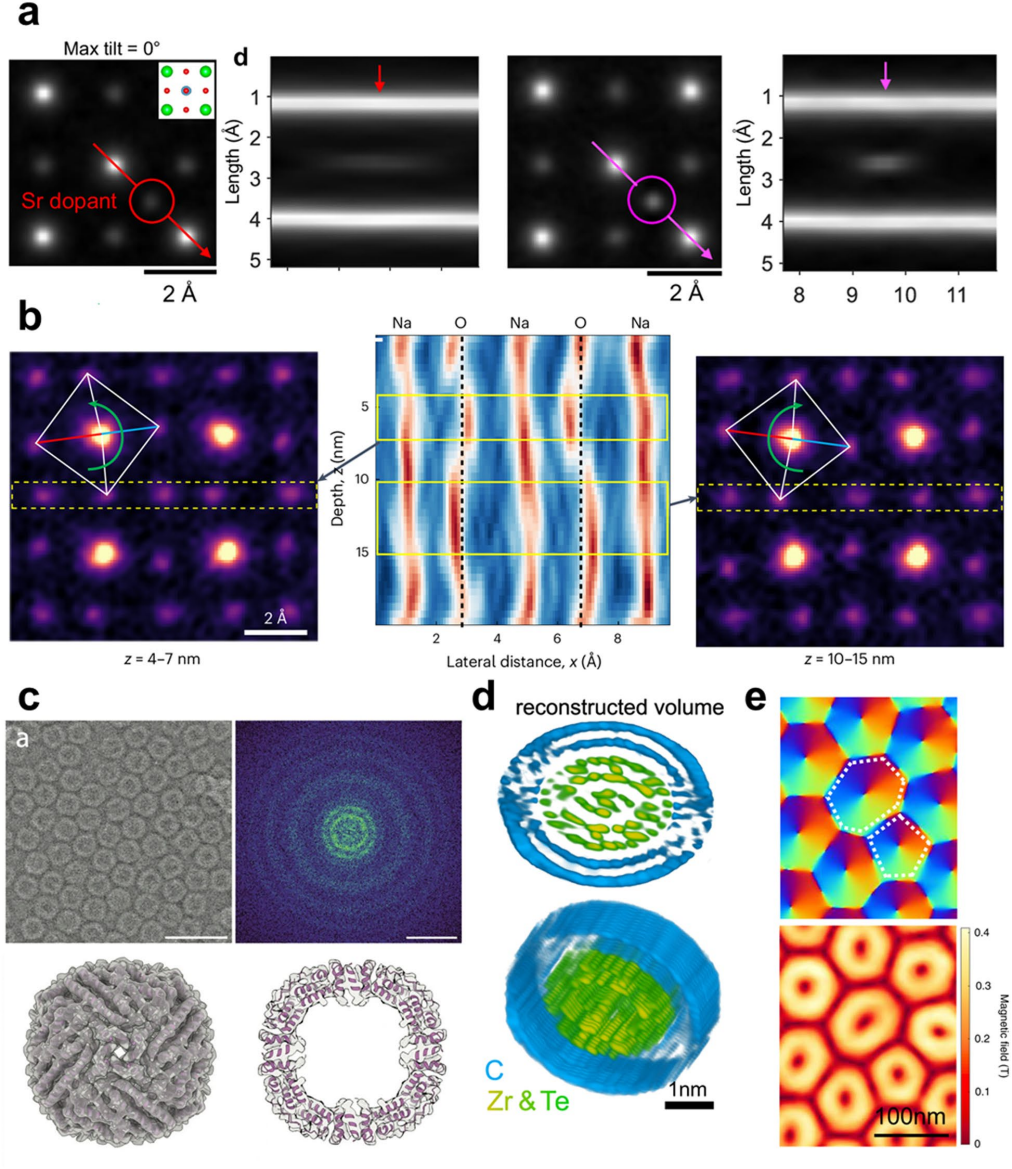
Examples showing advances in resolution by electron ptychography. **a** Reconstructed phase image of a slice containing a Sr dopant (marked by circles) in SrTiO_3_ crystal by tilt-coupled multislice electron ptychography and its depth profile. Data shown was acquired at maximum tilts of 0⁰ (left) and 4⁰ (right) (Dong et al. 2025). **b** Ptychographic images of NaNbO_3_ depth sectioning along the Na–O that show multiple nanoscale phases with distinctive octahedral rotations along different thicknesses (Kp et al. 2025). **c** The ptychographic image reconstruction of the Apoferritin protein, Fourier transformed image, 3D reconstructed image and its cross-section view, from left to right (Küçükoğlu et al. 2024). Scale bars are 30 nm. **d** Two orientations of the 3D reconstructed DW-CNT volume based on the 2D phase-contrast projection images reconstructed using mixed-state electron ptychography (Pelz et al. 2023). **e** Lateral magnetic field phase (above) and amplitude map (below) of skyrmions in FeGe near a dislocation core, determined from electron ptychography (Chen et al. 2022)

Based on algorithmic developments combined with instrumental improvements, electron ptychography can serve as a versatile technique across diverse fields including material science, physics, chemistry, and biology, enabling visualization of structures and properties previously inaccessible with conventional microscopy techniques. Due to its significant enhancement in resolution, various subtle structural features such as single dopants or vacancies can now be resolved. One example is visualization of single dopants in SrTiO_3_ crystal containing artificially introduced interstitial and substitutional dopants by tilt-coupled MEP (Dong et al. 2025). Figure 4a shows reconstructed phase images at the same depth for maximum tilt angles 0° and 4°, resolving a single Sr dopant (highlighted by circles). Increasing the tilt angle enhances the visibility of the dopant due to reduced depth blurring, precisely extracting the dopant location along the depth direction. Such depth-based analysis can further eliminate structural ambiguity in thick samples under strain, bias or effects from various defects within the sample that can locally alter the structure. For example, Harikrishnan et al. (2025) resolved distinctive ferroelectric distortions in NaNbO₃ with depth as in Fig. 4b, clearly capturing the structural differences in the bulk region from that of the relaxed surface area. Another advancement through electron ptychography is visualization of light elements and single vacancy detections in particular, which have been challenging due to weak scattering and low contrast. Since ptychography reconstructs the phase shift, it is sensitive to light elements. In this regard, electron ptychography can visualize boron-terminated tetravacancies in h-BN, resolving atomic distortions and bonding effects at defect edges (Byrne et al. 2504). Similarly, Dong et al. identified oxygen vacancies and self-doped ligand holes in La₃Ni₂O_₇-δ_, providing insights on the effect of nanoscale stoichiometry to superconductivity (Dong et al. 2024). Collectively, these studies highlight electron ptychography as a high-precision imaging technique capable of resolving subtle localized features at a sub-atomic scale for further insight into physical phenomena.

Beyond high-resolution imaging capabilities, electron ptychography serves as a particularly compelling method for imaging beam-sensitive materials which are often damaged under conventional STEM imaging conditions. This advantage stems from several factors, but most importantly from the fact that it reconstructs directly interpretable phase images with inherently higher contrast than Cs-corrected STEM. This enables high-resolution imaging at lower electron doses. Moreover, the use of a defocused probe in ptychographic acquisition distributes the beam over a larger area, effectively lowering the electron dose imparted to the sample and mitigating radiation damage. Zhang et al. (2023) demonstrated ultrahigh spatial resolution imaging of zeolites – typically considered beam-sensitive that can only tolerate a few thousand electrons per square angstrom –, successfully resolving individual oxygen atoms. The highest resolution previously achieved with STEM was approximately 1 Å (Shen et al. 2020). An example in chemistry is to image beam-sensitive core–shell upconverting nanoparticles that have fluorine atomic constituents. By utilizing electron ptychography, three-dimensional imaging over larger volumes than those accessible with atomic electron tomography (AET) were demonstrated to be possible in a single scan without causing significant beam damage. This allowed direct observation on tilts of the nanoparticle changing with depth, suggesting a twist of the shell structure that plays a critical role in determining their functional performance (Ribet et al. 2024). In biology, low-dose electron ptychography was demonstrated under cryogenic conditions to overcome the low SNR inherent in cryo-EM (Küçükoğlu et al. 2024). Various frozen-hydrated single particle protein samples including apoferritin (ApoF) protein was reconstructed in three-dimension at sub-nanometer resolution (Fig. 4c). This shows great potential for 3D reconstruction of biological tissue samples due to positive contrast transfer function (CTF) with zero-crossing, strong SNR, and volumetric visualization through multislice reconstruction.

By integrating electron ptychography with conventional tomography, it becomes possible to achieve three-dimensional imaging in high precision. Unlike traditional tomography—which often suffers from poor contrast for light elements and multiple scattering effects—ptychographic methods can provide additional depth sensitivity to overcome these limitations and offer enhanced resolution and elemental visibility. Such efforts are aligned with the recent advances in atomic electron tomography (AET), which has already demonstrated atomic-scale three-dimensional imaging and revealed the importance of improving image contrast and detection sensitivity for complex materials (Miao et al. 2016). In this domain, ptychographic atomic electron tomography (PAET) has been developed based on tilt series (Pelz et al. 2023) which corrects nonlinear effects arising from multiple scattering and enhances sensitivity to low-Z elements under significantly reduced electron dose conditions. Figure 4d shows an example of structure determination of complex double-wall carbon nanotube (CNT)-encapsulated Zr_11_Te_50_ structure in detail, observing a three-unit-cell-wide ZrTe_2_ structure, a new phase that was not observed before, being encapsulated by eight one-dimensional ZrTe_5_ clusters. Additionally, Schloz et al. (2025) introduced multi-focus electron ptychography, which enhances depth resolution by acquiring a defocus series within a 4D-STEM framework. This approach does not require tedious tilting procedures over a large range of angles and does not require complex reconstruction with respect to a fiducial as in 3D tomography, offering a more efficient and accessible alternative for three-dimensional analysis.

Another important application for electron ptychography is magnetic imaging, where resolving magnetic textures with high spatial resolution has long been a challenge. Imaging non-trivial topological textures from magnetic dipole signals has been achieved using DPC in Lorentz STEM with a theoretical resolution of sub-10 nm. However, the spatial resolution in Lorentz STEM is fundamentally limited by beam delocalization arising from defocused illumination. By combining a low-noise, high-dynamic-range detector with Lorentz STEM, Chen et al. (Chen et al. 2022) demonstrated that Lorentz electron ptychography (LEP) can reconstruct the phase induced by the magnetic flux from skyrmions (Fig. 4e). Using LEP, the internal structure of the skyrmion lattice was visualized through a field direction map with sub-nanometer resolution. Such real-space magnetic imaging can provide a valuable approach for probing the topology, dynamics, and singularities of spin textures in a range of magnetic materials and spintronic devices.

## Summary and outlook

This review highlights recent developments in 4D-STEM electron ptychography, focusing on algorithms, instrumental aspects, and applications. Algorithmically, a wide range of iterative and non-iterative methods has been developed to improve accuracy and robustness of phase reconstruction under experimental uncertainties. Advances such as MEP and mixed-state approaches have enabled ptychographic imaging of thick samples and partially coherent probes, broadening the technique’s applicability beyond ideally thin specimens. Recent improvements in pixelated detectors with high dynamic range, fast frame rates, and high DQE have enabled high-resolution electron ptychography to be more accessible under practical acquisition speeds and beam conditions. On the application side, electron ptychography has proven effective for visualization of locally varying structures, detection of light elements and vacancies with sub-nanometer depth resolution. Furthermore, its successful integration with complementary imaging techniques such as tomography and Lorentz microscopy suggests its versatility and potential to offer better insights into complex material systems.

Despite these recent advances, some challenges still remain to fully realize the potential of electron ptychography across diverse research fields. First, the loss of atomic number (Z) contrast poses unambiguity in compositional analysis. Unlike HAADF-STEM, where contrast is roughly proportional to Z^1.7^, contrast of reconstructed data does not show one-to-one correspondence with atomic number but shows high dependence on the outer shell valence of each element (Cao et al. 2018). While few studies have demonstrated distinguishability between similar-Z elements such as Cu and Zn (Denzer et al. 2025), achieving a straightforward and broadly applicable Z-sensitivity still remains a challenge. Second, while multislice electron ptychography has relaxed the strict thickness limits of traditional reconstruction, successful experimental reconstructions remain challenging in specimens thicker than a several tens of nanometers. This is because the probe undergoes multiple scattering in thick samples and is more strongly affected by aberration variations over a larger focal range, often leading to poor convergence or failure in reconstruction. Some recent efforts, such as applying energy filtering to limit inelastic scattering, have shown promise in extending the usable thickness range. Nevertheless, robust and reproducible reconstructions across a broad range of sample thicknesses remain an area of active development (Yang et al. 2025).

Looking forward, electron ptychography is expected to evolve beyond static 2D imaging and integrate with emerging STEM experimental schemes. If sufficiently fast acquisition speed and fidelity become available, innovative 4D-STEM acquisition schemes – such as in-situ microscopy, tomography and time-resolved scanning – could be combined with electron ptychography. For instance, the acquisition of a time series 4D-STEM datasets, also referred to as 5D-STEM or *k*-resolved electron correlation microscopy (ECM), has been developed to study time-evolving dynamics in nanomaterials (Huang and Voyles 2023). In parallel, although currently limited to optical systems, a method called time-resolved imaging via multiplexed ptychography (TIMP) has been proposed, where diffraction patterns from a series of pulsed illuminations are collected on a direct detector. Utilizing the known temporal separation between pulses, both the probe and the object can be ptychographically reconstructed frame by frame, thus enabling a “motion picture” of the sample dynamics (Barolak et al. 2024). These emerging experimental modalities suggest that combining ptychography with existing advanced imaging techniques could enable the study of dynamic processes with both structural and phase sensitivity, paving the way for a broader application of electron ptychography. As these approaches continue to develop and become more accessible, electron ptychography will serve as a highly versatile imaging method in modern STEM workflows across material science, physics and nanoscience.

## Data Availability

The dataset used during the current study are available from the corresponding author on reasonable request.

## Abbreviations

TEM: Transmission Electron Microscopy
HRTEM: High-resolution Transmission Electron Microscopy
STEM: Scanning Transmission Electron Microscopy
4D-STEM: Four-dimensional Scanning Transmission Electron Microscopy
Z: Atomic number
Cs-corrected: Spherical aberration-corrected
ADF: Annular dark-field
HAADF: High-angle Annular Dark-field
DPC: Differential Phase Contrast
MEP: Multislice Electron Ptychography
MTF: Modulation Transfer Function
